# Supplementary material for: Downregulation of Mitochondrial Fusion Protein Expression Affords Protection from Canonical Necroptosis in H9c2 Cardiomyoblasts
Source: Int J Mol Sci. 2024 Mar 2;25(5):2905. doi: 10.3390/ijms25052905 (PMC10932252; doi:10.3390/ijms25052905)
Supplement: Supplementary file 1 [file ijms-25-02905-s001.zip › ijms-2872700-supplementary.pdf]

International Journal of
Molecular Sciences

MDPI

Supplemental Figures

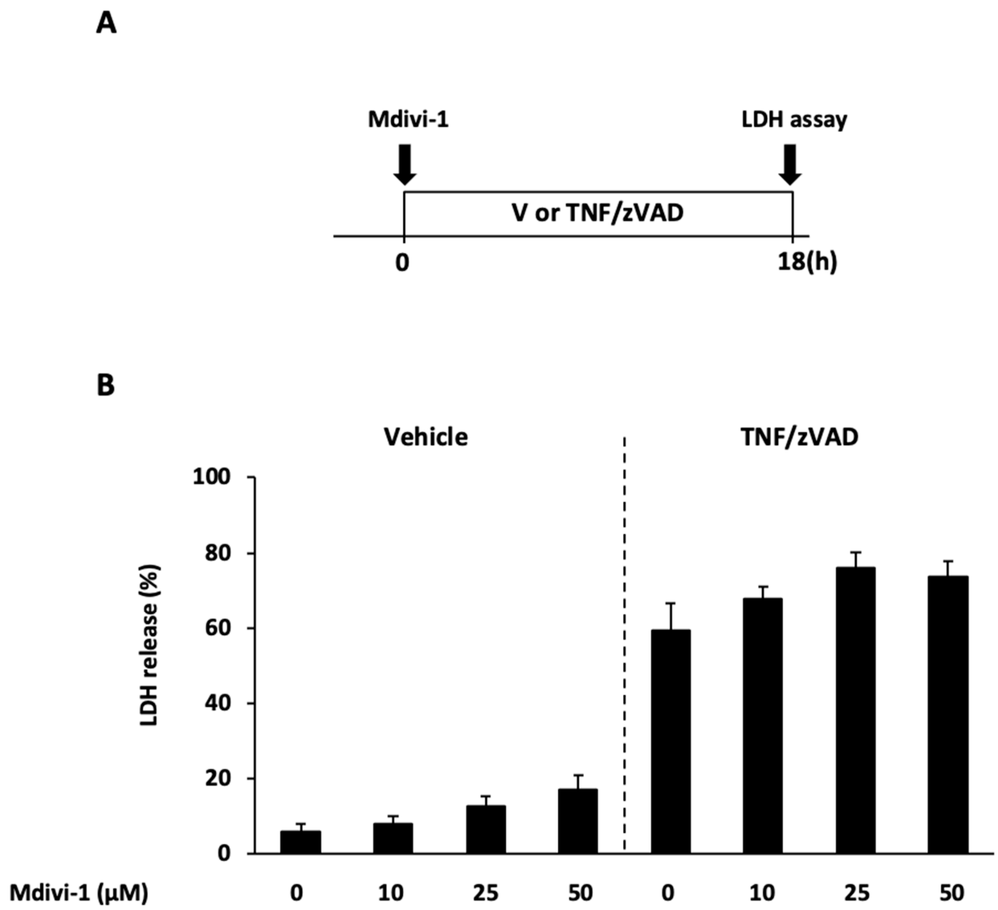

Figure S1. (A, B) Effects of Mdivi-1, a Drp1 inhibitor, on TNF/zVAD-induced LDH release. Experimental protocol (A) and results of quantitative analyses (B) are shown. H9c2 cells were treated with vehicle or various concentration of Mdivi-1 at the same time as the addition of vehicle or TNF-α and zVAD (TNF/zVAD; TNF-α, 50 ng/ml; zVAD, 20 μM). .

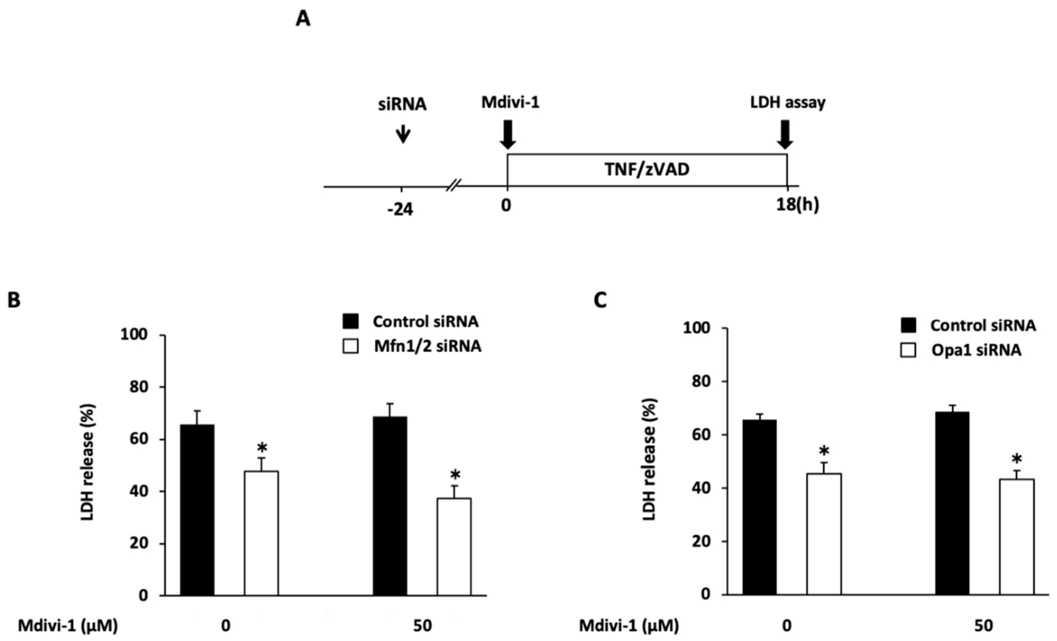

Figure S2. (A-C) Effects of Midivi-1, a Drp1 inhibitor, on protection from TNF/zVAD-induced cell death by knockdown of Mfn1/2 and Opa1. Experimental protocol (A) and results of quantitative analyses (B and C) are shown. The siRNAs were transfected into H9c2 cells 24 h before the addition of TNF-α and zVAD (TNF/zVAD; TNF-α, 50 ng/ml; zVAD, 20 µM). Mfn1/2 = mitofusin 1/2, Opa1 = optic atrophy-1. N = 4 in each group. *$p<0.05$ vs. cells transfected with control siRNA. .

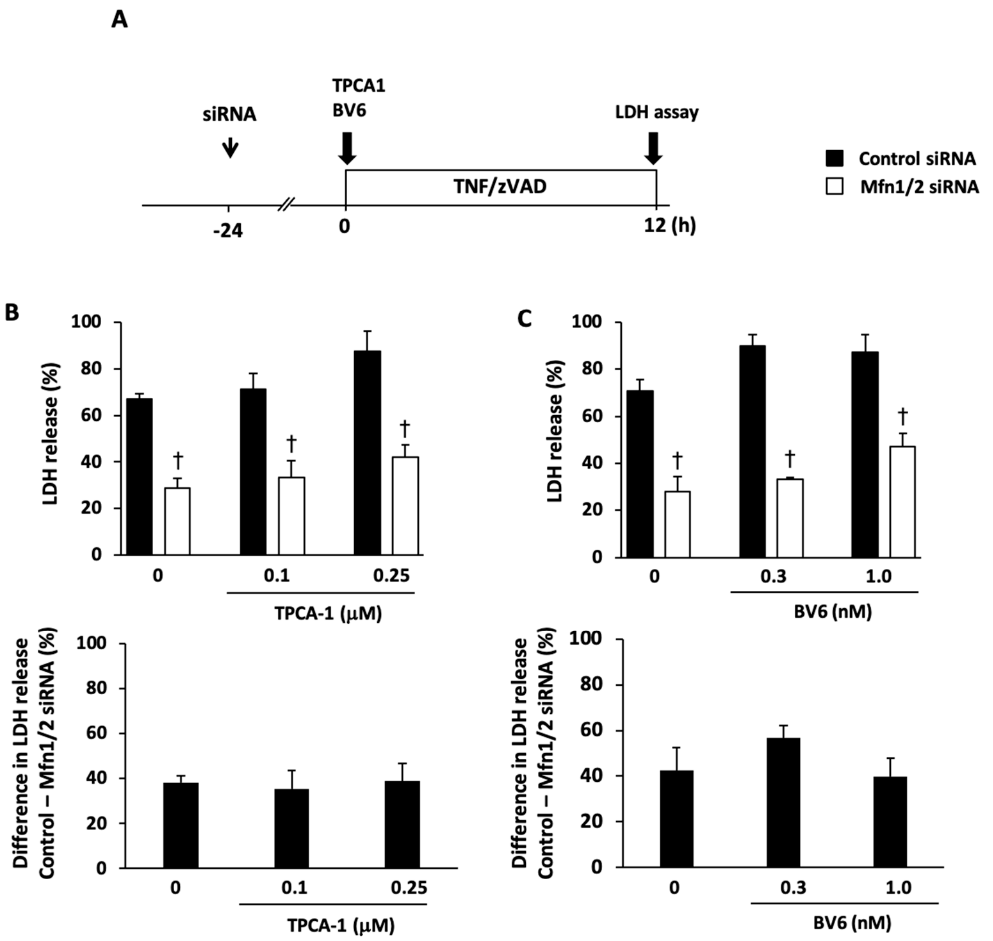

Figure S3. (A, B) Effects of TPCA-1, an IKKα/β inhibitor, and BV6, an inhibitor of cellular inhibitor of apoptosis proteins 1, on protection from TNF/zVAD-induced cell death by knockdown of mitochondrial fusion-regulating proteins. Experimental protocol (A) and results of quantitative analyses (B and C) are shown. The siRNAs were transfected into H9c2 cells 24 h before the addition of TNF-α and zVAD (TNF/zVAD; TNF-α, 50 ng/ml; zVAD, 20 μM). Mfn1/2 = mitofusin 1/2, Opa1 = optic atrophy-1. N = 6 in each group. †$p<0.05$ vs cells transfected with control siRNA and treated with similar concentration of inhibitors.

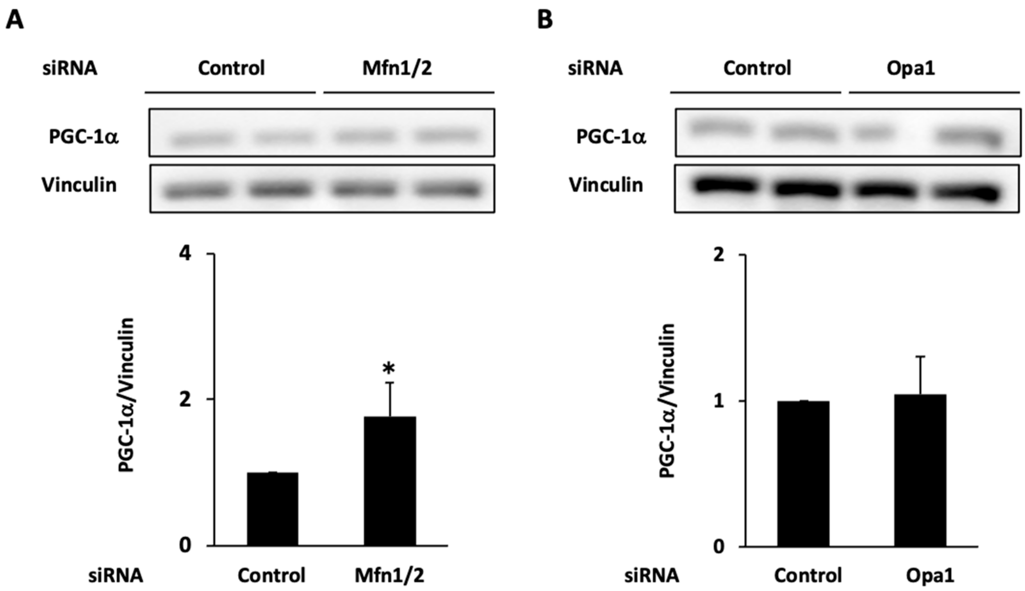

Figure S4. (A, B) Effects of knockdown of mitofusin (Mfn) 1/2 (A) and optic atrophy-1 (Opa1, B) on levels of peroxisome proliferator-activated receptor gamma coactivator-1 α (PGC-1α). Representative Western blots and results of densitometric analyses are shown. The siRNAs were transfected into H9c2 cells and cell lysates were collected 24 h after the transfection of siRNAs. N = 4 in each group. *$p<0.05$ vs. cells transfected with control siRNA.
